# Giving women WOICE postpartum: prevalence of maternal morbidity in high-risk pregnancies using the WHO-WOICE instrument

**DOI:** 10.1186/s12884-021-03727-3

**Published:** 2021-05-05

**Authors:** M. N. Lamus, S. Pabon, C. MPoca, J. P. Guida, M. A. Parpinelli, J. G. Cecatti, M. F. Vidarte, M. L. Costa

**Affiliations:** 1 Department of Obstetrics and Gynecology, School of Medicine, University of Campinas, Rua Alexander Fleming 101, Campinas, São Paulo, 13083-891 Brazil; 2 Department of Obstetrics and Gynecology, Foundation Valle del Lilli, Cali, Colombia

**Keywords:** Maternal morbidity, Mental health, Functionality, Depression, Puerperium, WHODAS 2.0, PHQ-9, GAD-7

## Abstract

**Background:**

There are no accurate estimates of the prevalence of non-severe maternal morbidities. Given the lack of instruments to fully assess these morbidities, the World Health Organization (WHO) developed an instrument called WOICE. We aimed to evaluate the prevalence of non-severe maternal morbidities in puerperal women and factors associated to impaired clinical, social and mental health conditions.

**Method:**

A cross-sectional study with postpartum women at a high-risk outpatient clinic in southeast Brazil, from November 2017 to December 2018. The WOICE questionnaire included three sections: the first with maternal and obstetric history, sociodemographic data, risk and environment factors, violence and sexual health; the second considers functionality and disability, general symptoms and mental health; and the third includes data on physical and laboratory tests. Data collection was supported by Tablets with REDCAP software. Initially, a descriptive analysis was performed, with general prevalence of all variables contained in the WOICE, including scales on anxiety and depression (GAD-7 and PHQ-9- impaired if ≥10), functionality (WHODAS- high disability scores when ≥37.4) and data on violence and substance use. Subsequently, an evaluation of cases with positive findings was performed, with a Poisson regression to investigate factors associated to impaired non-clinical and clinical conditions.

**Results:**

Five hundred seventeen women were included, majority (54.3%) multiparous, between 20 and 34 years (65.4%) and with a partner (75,6%). Over a quarter had (26.2%) preterm birth. Around a third (30.2%) reported health problems informed by the physician, although more than 80% considered having good or very good health. About 10% reported any substance use and 5.9% reported exposure to violence. Anxiety was identified in 19.8% of cases, depression in 36.9% and impaired functioning in 4.4% of women. Poisson regression identified that poor overall health rating was associated to increased anxiety/depression and impaired functioning. Having a partner reduced perception of women on the presence of clinical morbidities.

**Conclusion:**

During postpartum care of a high-risk population, over one third of the considered women presented anxiety and depression; 10% reported substance use and around 6% exposure to violence. These aspects of women’s health need further evaluation and specific interventions to improve quality of care.

## Background

To ensure a healthy life and promote well-being for all is among the new objectives of the Sustainable Development Goals for 2030, including the improvement of maternal health and reduction of maternal mortality. It has been suggested that, for each maternal death, 20–30 women suffer from some morbidity; however, these numbers are not based on standardized methods of assessment [1–3].

In the last decade, there has been important progress in the study of severe maternal morbidity (SMM), with standard criteria for the identification of potentially life-threatening conditions (PLTC) and Maternal Near Miss (MNM) [4]. Nevertheless, there is growing interest in understanding morbidity in a broader way, including non-severe morbidity. Non-severe morbidity are conditions that may influence and affect women’s health and well-being; they include impairment of women’s physical, sexual or mental health, and the ability to function in certain domains (cognition, mobility, participation in society), and also the image of their body and their economic and social status [5]. Most of these are not routinely evaluated in the clinical setting with potentially significant impact in women’s life.

In 2012, the World Health Organization (WHO) Maternal Morbidity Working Group (MMWG) initially developed a new definition of maternal morbidity as “Any health condition attributed to the complication of pregnancy and / or childbirth that may have a negative impact on the well-being and / or functionality of women” [1, 5]. The relevance of such definition is the innovation in capturing broadly the entire spectrum of morbidity, not excluding the well-known severe maternal morbidity conditions, but also including the non-severe morbidity as well [3].

The MMWG further developed an instrument called WOICE, to measure maternal morbidity, focusing on the health and well-being perception that women have about themselves [1, 6, 7]. The main purpose of this instrument is to identify women suffering of non-severe maternal morbidities, allowing professionals to give adequate care to those conditions, which may not be clearly identified during routine care. This instrument also standardizes the measurement of non-severe maternal morbidities, by using a common framework, allowing different settings and regions to share data and provide strong evidences during pregnancy and the postpartum period.

WOICE comprises tools already developed and validated in the literature, and the results of the pilot study have already been published [3, 6]. The pilot study using WOICE occurred in three different countries: Jamaica, Kenya and Malawi, between 2015 and 2016 [6] in pregnant women (around 28 weeks) and puerperium (between 6 and 12 weeks), mostly in centers with medical care for low risk pregnancies including a total sample of 1490 female participants (750 pregnant and 740 postpartum) [6]. It highlighted the high occurrence of non-severe morbidity in those countries, a condition not correctly identified in other studies that focused only severe maternal morbidity.

WOICE instrument is intended to give voice to neglected conditions in routine care. Lack of knowledge about such conditions lead to inadequate care of these women and contributes to possible short and long- term consequences. Those women who are neglected in the puerperium, return to their homes, with unidentified needs, thus impacting life with their family, newborn and spouse [8, 9].

WOICE represents a new approach towards measuring non-severe maternal morbidity, allowing health professionals to have a broader understanding of women beyond clinical diseases [1, 5].

The objective of the present study is to evaluate the prevalence of non-severe maternal morbidity among puerperal women and analyze factors associated with impaired clinical, social and mental health conditions in a middle – income setting using WOICE.

## Methods

This cross-sectional study used a questionnaire (which includes several instruments) developed by the WHO to assess maternal morbidity in its various aspects. The questionnaire was applied at the postpartum outpatient clinic of the University of Campinas, a public university hospital, in a single encounter with women from 6 to 12 weeks postpartum (scheduled for medical care and follow-up as routine care). This public health outpatient clinic is a referral center for women who delivered at the maternity hospital and cases scheduled include high-risk women, due to a clinical underlying condition or any complication diagnosed during pregnancy or childbirth. The maternity hospital is a referral center for women with conditions such as hypertension, diabetes, preterm labor and preterm rupture of membranes. Overall patients are from low-income background.

WOICE includes several tools that have already been previously translated and adapted to Portuguese. It includes the 12-item version of the World Health Organization’s Disability Assessment Schedule (WHODAS 2.0). This tool evaluates the functionality and ability to perform daily tasks [10–12].

WOICE also includes a tool that evaluates mental health, the General Anxiety Disorder 7-item test (GAD-7), and the 9-item Patient Health Questionnaire (PHQ-9), to assess depression, both already adapted to Portuguese [13, 14].

To measure substance use and abuse, WOICE includes Alcohol, Smoking and Substance Involvement Screening Test (ASSIST) [15, 16]. For sexual satisfaction and sexual and domestic violence, parts of some scores already validated are within the WOICE, such as the Brief Sexual Symptom Checklist for Women (BSSC-W) and some questions from a questionnaire used in the Multi-country Study on Women’s Health and Domestic Violence against Women of the WHO [17]. Only those last two tools were not previously validated into Portuguese versions, however they contributed with only 5 questions on a total of 126.

Finally, WOICE gathers data on woman’s background, current clinical symptoms and physical examination. The name of the tool precisely refers to the importance of not only consulting a woman during pregnancy and postpartum, but also of “listening” to her voice, complaints and needs.

The proposal was approved by the local Institutional Review Board. All women with age higher than 18 years that agreed to participate signed an Informed Consent form before interview. For adolescents (age bellow 18 years at the time of the interview), written consent and parental consent were both waived, due the consideration that a written consent and a parental consent could put the subject at risk, since violence is one of the conditions evaluated by the study, and it is well known that in cases of domestic violence, the perpetrator is often responsible or very close to the adolescent. However, interviews were conducted only after clarification and verbal consent, in a reserved room. The local Institutional Review Board of approved this procedure.

The maternal morbidity measurement questionnaire called WOICE was originally developed in English and further translated into the Brazilian Portuguese. The review was conducted by experienced obstetric investigators and the version was tested (pilot interviews) to measure the time of application and then adapt and modify some words to ascertain accurate understanding. In order to ensure the high quality and reliability of the information collected, the researchers were previously trained to ensure adequate use of the tablets and instruments included in the WOICE questionnaire.

Women were recruited sequentially according to their scheduled postpartum visit, during the data collection period (from November 2017 to December 2018). The postpartum outpatient clinic works every week day, a mean of 6 new cases/day, and they are scheduled according to availability. All women were invited and those who agreed to participate were interviewed. Sample size was estimated in 500 participants for convenience sample, as a pilot study, taking into account that the WOICE instrument had not been previously published by the time when data collection was initiated. The only previous study using such instrument presented 250 women during postpartum care (PPC) for each considered country [6].

Data collection was supported by tablets (Samsung Galaxy Tab Tablets S3 – Android), with further transmission, verification and storage of data protected to ensure confidentiality. Each interview was around 30–40 min. The questionnaire was always performed after the scheduled medical consultation and with no interference in the woman’s medical follow-up. Since some of the questions could potentially lead to unpleasant memories and reveal exposure to violence and substance abuse, additional support was always offered.

Data processing and collection were supported by REDCAP software and later transferred to the SPSS program. The information gathered was stored in a server located in the informatic department of the institution. A descriptive analysis was performed, including socio-demographic data, clinical and obstetric history, as well as the general prevalence of scores of instruments considered for functional and mental health. Continuous variables were presented on mean (M) and Standard Deviation (SD) and categorical variables in percentage (%) of frequency. An evaluation of abnormal conditions was performed, considering scores ≥10 for anxiety and depression [18, 19]. For WHODAS-12, according to a previous study published, dysfunctionality was considered with the score of ≥37.4 (95th percentile as the cutoff point) [10]. Missing data was described in tables.

Further, a Poisson multiple regression analysis was performed, providing the respective Prevalence Ratio (PR) and 95% confidence intervals (CI), considering three models for evaluating factors associated with impaired conditions. Predictors were chosen considering those with a *p*-value lower that 0.05 and excluding variables that are highly correlated (we included education level and not illiteracy). The first model considered as outcome abnormal mental health (score ≥ 10 for anxiety and depression questionnaire), the second model considered abnormal functioning. The predictors tested were: maternal age, marital status, education,, employed, travel time to facility, parity, gestational age, BMI (≥30 kg/m2), overall health rating, any clinical condition, preexisting conditions, taking any medication. The third model of logistic regression considered impaired clinical condition (women who answered “yes” to the question: “have you been told you have anything wrong or any medical condition?”) as outcome. The tested predictors were the same used in the previous models, also in addition to impaired mental health, abnormal functioning, substance use, sexual satisfaction and violence.

## Results

**Fig. 1:**
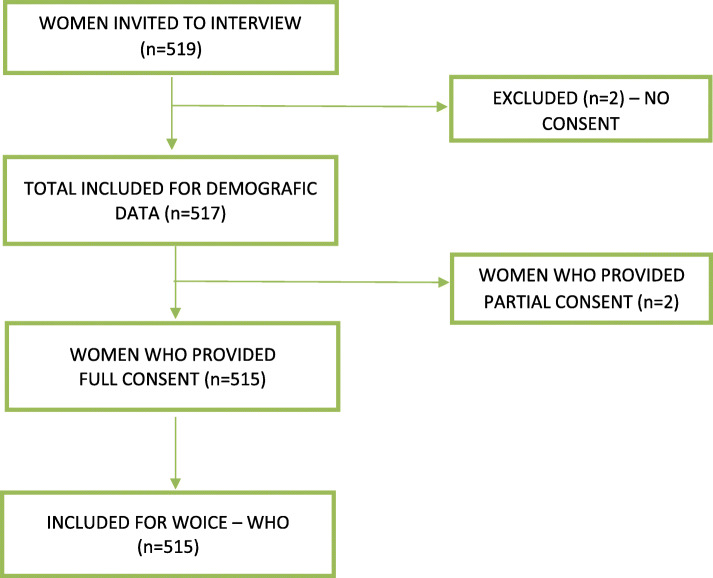
Flowchart of postpartum women included in the study

In the present study, 519 postpartum women were invited to participate, 2 declined and the 2 women provided only sociodemographic data, therefore 515 gave full consent (Fig. 1). The mean age was 28 years, women mostly had a partner, more than 50% were multiparous, the illiteracy level was less than 2.4% and most participants had a secondary level of education and were employed. Over one third of the population took 30–60 min to arrive from their house to the health service (Table 1).

**Table 1:** Sociodemographic and obstetric characteristics of postpartum women (*n* = 517)

	Characteristic	PPC	%
		*N* = 517	
	Mean (SD)	28.3 (±7.0)	
Maternal age	< 20	66	12.7
	20–34	338	65.4
	> 34	113	21.9
Marital Status^a^	No partner	126	24.4
	Has partner	390	75.6
Education^b^	Primary or less	97	18.8
	Secondary	343	66.5
	Higher	76	14.7
Literacy^c^	Cannot read	4	0.8
	Can read parts of sentence	8	1.5
	Can read whole sentence	504	97.7
Employed	No	206	39.8
	Yes	311	60.2
Travel time to facility, minutes^d^	< 15	64	12.4
	15–30	167	32.4
	30–60	190	36.8
	> 60	95	18.4
Parity ^e^	1	234	45.8
	2 to 4	261	51.1
	> 5	16	3.1

Missing information - a: (1), b: (1), c:(1), d: (1), e: (6)

Clinical conditions were initially considered through the question: “Since childbirth, have you been informed that there is something wrong / some medical condition?” and 30.2% of the women had a health condition reported by the attending physician, although more than 80% reported good or very good health. Considering the gestational results, a quarter (26.2%) had preterm birth, and 58.3% delivered by cesarean section; however, predominantly with good perinatal outcomes, 95.7% reported “good baby health” in the postpartum evaluation, with 88.1% of exclusive breastfeeding (Table 2).

**Table 2:** Perinatal outcomes, clinical conditions and overall conditions considered by the WHO- WOICE among postpartum (PPC) women (*n* = 515)

Variable	PPC^#^ *N* = 515	%
Preterm delivery ^a^		
No	364	73.8
Yes	129	26.2
Healthy baby ^b^		
No	17	3.5
Yes	469	95.7
Unknown	4	0.8
C-section ^c^		
No	213	41.7
Yes	298	58.3
Breastfeeding ^d^		
No	59	11.9
Yes	435	88.1
Overall health rating ^e^		
Very good	119	23.3
Good	305	59.7
Neither poor nor good	67	13.1
Poor	18	3.5
Very poor	2	0.4
Have you been told you have anything wrong /any condition (s)? ^f^		
No	353	69.8
^Yes^	153	30.2
Are you taking any medication(s)?		
No	314	61.0
Yes	201	39.0
Dou you have any other medical condition or the other problem (s) you would like to report? ^g^		
No	249	49.0
^Yes^	259	51.0
Any preexisting conditions		
No	249	48.3
Yes	266	51.7
Leading direct preexisting conditions		
Gestational diabetes	71	13.8
Gestational hypertension	69	13.4
Pre-eclampsia	55	10.7
Urinary tract infection	14	2.7
Premature preterm rupture of membranes	3	0.6
Others	10	2.2
Leading indirect preexisting conditions		
Chronic hypertension	42	8.2
Mellitus diabetes	16	3.1
Surgical wound infection	9	1.7
Syphilis	8	0.6
Others	27	5.4
Any condition diagnosed on interview day ^h^		
No	452	92.2
Yes	38	7.8
Number of conditions diagnosed**		
0	11	4.0
1	145	53.1
2	71	26.0
3	26	9.5
> =4	20	7.3

*Missing information* a: (22), b: (25), c: (4) d: (21) e: (4), f: (9), g: (7), h: (25)

^#^PPC- women in postpartum care

**n = 273. Women with no missing information for any of the considered instruments (considering 2 conditions diagnosed, 59 women presented anxiety and depression and 36 women presented any clinical condition and anxiety)

Looking into detail in cases of clinical conditions, based on the question: “any pre-existing condition”, the majority (51.7%) reported having a condition before pregnancy and childbirth (Table 2). A list of conditions, classified them as direct and indirect, of which 13.8% had gestational diabetes, followed by gestational hypertension (13.4%), preeclampsia (10.7%), chronic hypertension (8.2%) and, operative wound infection (1.7%), as the most prevalent types of diseases (Table 2).

An important approach, besides reporting pre-existing conditions, was to evaluate the amount of abnormal conditions diagnosed or identified by WOICE, when considering women that had answered all instruments (*n* = 273). We found that (53.1%) had at least one abnormal condition identified by WOICE, a quarter of women (26%) had two concomitant conditions identified by WOICE and only 4.0% had no abnormal condition. Among cases with two identified abnormal conditions, most common associations were anxiety and depression, and any reported clinical condition and anxiety (Table 2).

We identified, through the WOICE questionnaire in this group of women, the use of substances, asking participants whether they used (cigarettes, alcoholic beverages, marijuana, inhalants, sedatives or sleeping pills, hallucinogens, opioids and/or injectable drugs for non-medical use) and 10.0% of the participants used some type of substance during pregnancy (Table 3). In this group of questions, we also asked “during pregnancy, someone (friend, relative or anyone) expressed concern about the use of any substance” and 66.7% expressed such concern, followed by 50% of women that “tried to reduce or stop consumption of any substance”.

**Table 3:** Social and sexual conditions among postpartum women

	Variable	PPC	%
		*N* = 515	
*Substance use ^a^	No	460	90.0
	Yes	51	10.0
Damage in the day to day due to the use of substance (*N* = 51)			
	No	41	80.4
	Yes	10	19.6
Legal, social or financial problems due to the use of substance (*N* = 51)			
	No	48	94.1
	Yes	3	5.9
Relatives concern regarding the use of substance (*N* = 51)			
	No	17	33.3
	Yes	34	66.7
Tried to stop but couldn’t (*N* = 51) ^b^			
	No	25	50.0
	Yes	25	50.0
**Exposure to violence ^c^	No	477	94.1
	Yes	30	5.9
***Exposure to sexual violence^d^	No	490	99.2
	Yes	3	0.6
	Refused to answer	1	0.2
Sexual life after delivery ^e^	No	345	67.5
	Yes	166	32.5
Mean time after delivery for returning sexual activity (weeks)		5.08 ± 1.75	
Satisfaction with sexual life *N* = 166	No	18	10.8
	Yes	148	89.2
Reason of sexual unsatisfaction	Pain during sex	10	55.6
*N* = 18	Little or no interest in sex	9	50.0
	Decreased vaginal lubrication (dryness)	4	22.2
	Problems reaching orgasm	1	5.6
Use contraceptive method ^f^	No	310	61.1
	Yes	197	38.9
Prescription of contraceptive method on the interview day ^g^	No	111	22.8
Yes	Yes	375	77.2

Missing information* a: (4), b (1), c (8), d (21) e (4), e (3), f (8), g (29)

* Defined as use of the following substances: tobacco products, alcoholic beverages, marijuana (ganja), inhalants, sedatives or sleeping pills, hallucinogens, opioids and / or injectable drugs for non-medical use

** Women who responded no or never to the following question:

Since the delivery, was there ever a time when you were pushed, slapped, hit, kicked, or beaten by (any of) your husband/partner(s) or anyone else?

*** Women who responded no to the following question:

Since the delivery, has your current husband/partner ever forced you to have sexual intercourse when you did not want to, for example by threatening you or holding you down? OR Since the delivery, did you ever have sexual intercourse you did not want to because you were afraid of what your partner/husband might do if you refused? OR Since the delivery, did your husband/partner ever force you to do anything else sexual that you did not want or that you found degrading or humiliating?

Around 1/3 of women had already resumed their sex life after giving birth and 89.2% felt they were satisfied with their sex lives, however 55.6% (*n* = 10) reported pain during intercourse (Table 3). Around 39% of the women used contraception and 77.2% of them were prescribed with a method during their first postpartum care medical visit.

Using the WOICE tool, tool, we explored exposure to domestic and sexual violence by asking participants “whether or not they were afraid of the current partner / most recent spouse or any other person” if the spouse / or any other person who pushed, hit and kicked”. In our sample, 5.9% reported to have suffered violence (Table 3).

As part of the Mental Health assessment of our study, we used the validated scales (PHQ-9 and GAD-7). Abnormal conditions were considered if scores ≥10 [17, 18] and almost 20% of the women had anxiety symptoms, followed by 36.9% with depressive symptoms. For the evaluation of functionality or ability to perform daily tasks, used WHODAS-12 version 2.0 and verified that the mean score was 10.9 (±12.9), we found 4.4% of the women had high disability scores (score ≥ 37.4) [20]. (Table 4).

**Table 4:** Mental and functional conditions of the study population

Variable	PPC	%
	*N* = 515	
(a) Anxiety score Mean (SD)	5.5 [±5.4]	
Score ≥ 10	101	19.8
Score < 10	410	80.2
(b)Depression Mean (SD)	8.4 [±6.0]	
Score ≥ 10	73	36.9
Score < 10	125	63.1
(c) WHODAS −12 Mean (SD)	10.9 [±12.9]	
Score < 37.4	474	95.6
Score ≥ 37.4	22	4.4

(a) GAD-7: seven items, with four-point scale: 0 (not at all) to 3 (Several days). A score ranging from 0 to 21 is considered a positive indicator for anxiety, equal to or greater than 10 [10].

(b) PHQ-9: nine items, with a four-point scale: 0 (not at all) to 3 (several days), The score ranging from 0 to 27. It is estimated, as a positive indicator of major depression, equal to 10 [20]

(c) WHODAS 12. Contains 12 items, the scores of each question were recoded and later the following formula was used [20]: Compute S1-S12 = (S1 + S2 + S3 + S4 + S5 + S6 + S7 + S8 + S9 + S10 + S11 + S12) * 100/36

Among the included women, 28.3% used such support, of those 97% psychological support and 6.6% social service support (Table 5).

**Table 5:** Distribution of the referral for social, psychological or medical support after WOICE questionnaire

Variable		PPC	%
		*N* = 508	
Referral^a^	No	387	76.2
	Yes	121	28.3
Psychological		118	97.5
Medical		1	0.8
Social service		8	6.6

**missing information** ^a^**7**

In order to investigate factors independently associated with impaired functioning, mental health and clinical conditions, we performed three multiple regression analyzes. For the first model, that considered WHODAS≥37.4 as the outcome, the condition independently associated with abnormal functioning was the presence of impaired clinical health. Nevertheless, less education and having a partner were protective conditions towards the report of impaired functioning (Table 6). In model 2, considering as outcome abnormal anxiety and depression (scores ≥10), poor overall health rating was associated with increased anxiety/depression. However, increased parity was protective.

**Table 6:** Factors associated with alterations in functionality (model 1), in mental health (model 2) and clinical health (model 3) - Multivariate analysis

Model/ Variable	PR	IC 95% p/ PR	*p*
(a)Model 1 WHODAS≥37.4 [n=494]			
**Education (Primary or less)**	<0.01	<0.01 – <0.01	<0.001
**Overall health rating (Neither poor nor good; poor; very poor)**	11.96	4.87 – 29.39	<0.001
**Marital status (With partner)**	0.44	0.21 – 0.94	0.034
(b)Model 2 Anxiety / Depression [n=217]			
**Overall health rating (Neither poor nor good; poor; very poor)**	1.65	1.31 – 2.08	<0.001
**Parity (≥2)**	0.73	0.56 – 0.96	0.027
(c)Model 3 Clinical health [*n*=501]			
**Exposure to violence**	1.73	1.13 – 2.64	0.012

**Multiple analysis by Poisson regression**

(a) For model 1 the outcome was: WHODAS≥37.4 and the predictors were the variables: maternal age, marital status, education, employment, travel time to facility, parity, overall health rating, any clinical condition, preexisting conditions, taking any medication, categorized Body Mass Index (BMI) and mode of delivery.

(b) For model 2 the outcome was Anxiety score> 10 and Depression score> 10, And the predictors were the variables: maternal age, marital status, education, employment, travel time to facility, parity, overall health rating, any clinical condition, preexisting conditions, taking any medication, categorized Body Mass Index (BMI) and mode of delivery.

(c) For model 3 the outcome was any clinical condition reported by the woman and the predictors were the variables: maternal age, marital status, education, employed, travel time to facility, parity, BMI (≥30 kg / m2), alteration in mental health (Anxiety score≥ 10), sexual dissatisfaction, WHODAS≥37.4, exposure to domestic or sexual violence and use of substances.

In model 3, the clinical conditions reported by the women (defined when the woman reported having been informed of a clinical diagnosis after delivery) were considered as outcomes. We identified that the variable evaluated in the questionnaire about violence, as: “whether or not they were afraid of the current partner / most recent spouse or any other person”, was positively associated to the perception of impaired clinical condition (Table 6).

## Discussion

This study represents the continuation of an initiative led by the WHO Maternal Morbidity Working Group (MMWG), and represents the implementation the WOICE 2.0 questionnaire to measure non-severe maternal morbidity for the postpartum women considering a broad approach of conditions that can impact maternal health, in a high-risk setting [7].

The pilot study conducted in Jamaica, Kenya and Malawi tested the WOICE in pregnant and postpartum women, for the first time, in a mostly low risk and low-income settings, with a total sample of 1490 women [6]. In comparison to their findings, our sample included older, more educated women and mostly women with partners. In the pilot study, (6.1%) of the women reported having a health problem informed by the attending physician and in our study, this number was much higher, (over 50%), with more C-section and preterm birth.

Cesarean section rates are increasing worldwide, with Brazil among the most impressive figures (over 50%) [21, 22]. Our sample represents a referral center and there is possible selection bias through postpartum scheduled visits, since mostly complicated cases are the ones followed at the institution, therefore not representing the overall cesarean rate in the institution.

Another marker of high-risk assessment is the rate of prematurity. Preterm birth is the main risk factor for infant morbidity and mortality, not only during the neonatal period but also in childhood, it can affect the cognitive dimensions, physical health and behavior, so it is one of the most important challenges for public health. Brazil has rates of preterm birth around 11.5% [23].

We evaluated the exposure to violence in the WOICE questionnaire, where we could identify that in this group of women surveyed, 5.9% of the participants were exposed to some type of violence (domestic-sexual). Previous reports showed exposure to domestic violence against women as a global phenomenon and these victims are frequently very familiar with their perpetrators, who are people of their daily life. This violence is accepted as “normal” in many societies of the world [24]. Estimates by the WHO say that 1 in 3 women worldwide suffer from physical and / or sexual partner and sexual violence by third parties at some point in their life [25] Violence is a sensitive subject, since women are often afraid to talk about it, because of the possible repercussions. Our findings with low frequency of violence, might reflect such fear of the truth.

In Brazil, physical, sexual and psychological violence against women are gaining awareness with increase in legal protections and enhanced tools for reporting agressors. Data suggest that it has always been a major hidden problem in the country. From 2011 to 2017, almost half a million cases of intimate partner violence against women were registered in a national database of surveillance. Among pregnant women, data is scarce, and a recent study obtained similar rates of physical and sexual violence as ours (12.1 and 2.8%) [26, 27]. We believe that violence against women is underreported and an adequate surveillance is mandatory to understand the dimension of the problem and to propose national policies to guarantee the needed support.

The high frequency of breastfeeding in our sample must be highlighted, especially considering the high-risk background and frequency of prematurity. Studies show that one of the priorities of these women is the good development of the baby that is supplied in large part by the mother’s milk, thus reducing early weaning [28], this might support such levels of breastfeeding, adding the hospital’s active work in campaigns, programs to inform women about the benefits of breastfeeding for the baby.

According to a study carried out in 2017, on the indicators of breastfeeding in Brazil in the last three decades, they have led Brazil to be considered a successful country in the implementation of policies and programs to promote breastfeeding with all the necessary tools, knowing that the breastfeeding is not only the responsibility of women, it is also shared with society. The prevalence of exclusive breastfeeding for children under 6 months of age in 2013 was 52.1% [20].

When considering abnormal conditions evaluated by the WOICE instrument, it was striking to observe less than 5% of women with no morbidities. This supports the understanding of multiple aspects that are able to influence women’s wellbeing and that during postpartum, women need multidisciplinary support. As a limitation, we do not have prospective assessment of women, in order to pursue the real impact of gestation throughout the reproductive cycle.

Poisson regression presented that having a partner decreased the women’s perception of clinical morbidities and functionality impairment; that might just reflect more care and support. Primary education (or less) was a protective condition towards functionality impairment evaluated by WHODAS. The underlying explanation for such finding is not clear yet and needs further studies, however, could represent the decreased ability to report or even less awareness towards the evaluated conditions in the WHODAS instrument. Having a clinical diagnosis was an independent factor associated to impaired mental health and functioning. This is expected, but rarely reported in a systematic way. Knowing that clinical conditions can be associated to further impairment can guide interventions and improve care [29] In our sample, there was a significant number of women with complications due to hypertension. It is important to highlight that preeclampsia and eclampsia are major causes of morbidity and mortality, especially in low and middle-income settings [30, 31].

It is important to note that 96% of women reported at least one morbidity evaluated by the WOICE instrument, during pregnancy or postpartum period. Performing regular care, we are most likely underreporting the occurrence of morbidities, if we consider the current WHO maternal morbidity framework. WOICE strengths the need to give voice to women during care: if we do not actively ask, we probably will not diagnose non-clinical and non-severe morbidities. However, if we really want to understand in depth the burden of maternal morbidity, we have to apply instruments that may bring to surface some underlying conditions, during antenatal care [32] and postpartum.

Those conditions may be extremely harmful to women, such as intimate partner violence or substance abuse. However, due to social stigmas, those conditions may be source of shame and not reported in routine care; we cannot consider that a woman with those conditions will undergo a positive pregnancy experience, and we will only conduct it properly if we ask.

Another interesting point of our results is that the majority of women reported good or very good health at the time of the interview. Our study design does not allow us to affirm any cause-consequence relation, however we suppose that such result is a consequence of the perception of good healthcare. Some morbid conditions may have occurred and since solved through the puerperium period. The study was performed in a referral center for high risk pregnancies. Women with underlying medical conditions, are frequently under increased clinical surveillance during pregnancy and postpartum and motivated to adhere to treatment because of fetal health. Therefore, many times they feel they are in “good health” and we hypothesize that such answer is a consequence of adequate healthcare.

An important concern regarding our results is that our sample represents a population attended in a high risk setting, and results may not be generalizable for the general obstetric population, or even those followed in low-risk settings. However, it highlights the importance of not only considering clinical morbidities, but also other morbidities, even in women with known underlying disease.

Postpartum care (PPC) would need to provide much more than contraceptive method orientation, it needs to ensure the opportunity to promote women’s health and well-being, and postpartum visits should include a thorough assessment of physical, social, psychological and mental health [9].

A relevant limitation is that the WOICE has not been translated and validated into different languages, as Portuguese, and it may difficult comparisons with data obtained using the English version. However, the tool is based on several instruments that have been previously validated, and this should be considered when analyzing its results. Another limitation is that questionnaires were answered through an interview administered by a researcher. This methodology may underreport the occurrence of morbidities, notably drugs consumption and intimate partner violence, however such approach was considered to allow the inclusion of women with low-education level.

More research and studies are needed with this instrument to validate it globally, identifying problems and conditions that are not evaluated in a common medical consultation, improving care for women after childbirth.

## Conclusions

The WOICE-WHO instrument allows for an overall evaluation of maternal morbidity. During postpartum care, women presented high frequency of anxiety and depression and relevant frequency of substance use and violence. These aspects of women ´s health need further evaluation and specific interventions to improve quality of care.

## Data Availability

The datasets used and/or analyzed during the current study are available from the corresponding author on reasonable request.

## Abbreviations

ANC: Antenatal care
ASSIST: Alcohol, smoking and substance involvement screening test
BSSC-W: Brief sexual symptom checklist for women
GAD-7: General anxiety disorder 7
MNM: Maternal near miss
MMWG: Working group maternal morbidity
PHQ-9: Patient health questionnaire 9
PLTC: Potentially life- threatening conditions
PPC: Postpartum care
PR: Prevalence ratio
REDCAP: Research electronic data capture
SD: Standard desviation
SMM: Severe maternal morbidity
SPSS: Statistical package for the social sciences
WHO: World health Organization
WHODAS: World health Organization disability assessment schedule 2.0
